# Functional Characterization of *PmDXR*, a Critical Rate-Limiting Enzyme, for Turpentine Biosynthesis in Masson Pine (*Pinus massoniana* Lamb.)

**DOI:** 10.3390/ijms25084415

**Published:** 2024-04-17

**Authors:** Rong Li, Lingzhi Zhu, Peizhen Chen, Yu Chen, Qingqing Hao, Peihuang Zhu, Kongshu Ji

**Affiliations:** 1 State Key Laboratory of Tree Genetics and Breeding, Nanjing Forestry University, Nanjing 210037, China; hblxylr@126.com (R.L.); lingzhi_zhu@126.com (L.Z.); pei_jane@126.com (P.C.); chenyu516106@126.com (Y.C.); hqq@njfu.edu.cn (Q.H.); phzhu@njfu.edu.cn (P.Z.); 2 Key Open Laboratory of Forest Genetics and Gene Engineering of National Forestry and Grassland Administration, Nanjing 210037, China; 3 Key Laboratory of Forestry Genetics & Biotechnology of Ministry of Education, Nanjing Forestry University, Nanjing 210037, China; 4 Co-Innovation Center for Sustainable Forestry in Southern China, Nanjing Forestry University, Nanjing 210037, China

**Keywords:** terpenoids, *Pinus massoniana* Lamb., 1-deoxy-D-xylulose-5-phosphate synthase, abiotic stresses, functional identification

## Abstract

As one of the largest and most diverse classes of specialized metabolites in plants, terpenoids (oprenoid compounds, a type of bio-based material) are widely used in the fields of medicine and light chemical products. They are the most important secondary metabolites in coniferous species and play an important role in the defense system of conifers. Terpene synthesis can be promoted by regulating the expressions of terpene synthase genes, and the terpene biosynthesis pathway has basically been clarified in *Pinus massoniana*, in which there are multiple rate-limiting enzymes and the rate-limiting steps are difficult to determine, so the terpene synthase gene regulation mechanism has become a hot spot in research. Herein, we amplified a *PmDXR* gene (GenBank accession no. MK969119.1) of the MEP pathway (methyl-erythritol 4-phosphate) from *Pinus massoniana*. The DXR enzyme activity and chlorophyll a, chlorophyll b and carotenoid contents of overexpressed *Arabidopsis* showed positive regulation. The *PmDXR* gene promoter was a tissue-specific promoter and can respond to ABA, MeJA and GA stresses to drive the expression of the *GUS* reporter gene in *N. benthamiana*. The DXR enzyme was identified as a key rate-limiting enzyme in the MEP pathway and an effective target for terpene synthesis regulation in coniferous species, which can further lay the theoretical foundation for the molecularly assisted selection of high-yielding lipid germplasm of *P. massoniana*, as well as provide help in the pathogenesis of pine wood nematode disease.

## 1. Introduction

Pine wilt disease (PWD), native to North America, is the most dangerous and devastating epidemic forest disease caused by pine wood nematode (PWN) [1,2]. PWN has spread to 701 county-level administrative regions and 5250 townships, with an epidemic area of up to 1,5115,13.3 hm^2^ in China (as of 2022). Masson pine (*Pinus massoniana* Lamb.) belongs to the *Pinus* subgen, Oleifera Group (*Pinus* sect. *Pinus*) of the genus *Pinus* (Pinaceae), which has a unique position in ensuring ecological security and timber resource supply in China [3]. China is the largest producer of tallow turpentine in the world, and according to statistics, Guangxi Province ranks first in the country in terms of tallow turpentine production year round, of which 80% of production comes from *P. massoniana* [4]. As a natural resource, the utilization of pine resin has immeasurable development prospects in the context of sustainable development and ecological protection. Cultivating excellent lipid-producing trees through genetic improvement is the only way to achieve sustainable development. Obviously, like *P. thunbergii* and *P. densifloras*, the most widely distributed *P. massoniana* in China is also facing a serious threat of high or moderate susceptibility [5]. The various secondary metabolic pathways in plants have mostly evolved over time in response to various adversities such as environmental changes and biotic stresses [6]. In PWD development, the role of host secondary metabolites and volatiles on PWN behavior may directly determine the rate of disease spread, such as the products of the phenylpropane pathway (i.e., phenolics) and the isoprene-like pathways (i.e., terpene resins), which have a strong killing effect on invasive organisms [7,8]. A good example is that pine resin can be released from traumatic resin ducts, while new pine resin can be synthesized by induction and local terpene reactions are released [9].

Early land plants and higher plants have a complete MEP (methyl-erythritol 4-phosphate) pathway and MVA (mevalonate-independent) pathway, which contain more than 1000 terpene secondary metabolites [10]. The terpene composition of *P. massoniana* pine resin consists mainly of monoterpenes, sesquiterpenes and diterpenes, which are generally stored in the roots, stems, needles and cones of pine trees. The monoterpenes (C_10_) in gymnosperms (especially pine and fir), mainly in the form of α-pinene, β-pinene, limonene and cotyledonene, are toxic to various insects [11]; sesquiterpenes (C_15_) can act as herbivore inhibitors and antifungal agents [12]; diterpenes (C_20_) prey on insects by exuding when natural enemies feed on the resin ducts [13]; azadirachtin, a triterpene (C_30_), has an extremely strong food-repelling and reproductive-growth-inhibiting effect on lepidopteran insects [14]; and polyterpenoids have protective mechanisms against oxidation, herbivores and wound healing [15]. It has been shown that β-pinene content in *P. massoniana* is positively correlated with host disease resistance [16,17]. The synthesis of most terpenoids relies on the MEP pathway. DXR (1-deoxy-D-xylulose-5-phosphate-reductoisomerase) catalyzes the conversion of DXP to MEP by a reduction reaction assisted by divalent metal ions (e.g., Mn^2+^, Co^2+^ or Mg^2+^) and the oxygen donor NADPH, which is the branching point of the “carbon flow” in the MEP pathway [18] and is also the most effective regulatory site. DXRase is a homodimer with an N-terminal transit peptide that directs DXR localization to the plasmid and has a conserved Cys-Ser-(Ala/Met/Val/Thr) motif; it has a proline-rich region P(P/Q)PAWPG(R/T). They have been successfully cloned from gymnosperms, such as *P. densiflora* [19], *Ginkgo biloba* [20], *Taxus chinensis* [21], and *T. madia* [22], and are commonly present as single-copy genes. Previous studies have shown that the overexpression of *DXR* genes in plants affects the terpenoid content. For example, it increased DXR activity and essential oil biosynthesis in leaf tissues of peppermint (*Mentha* × *piperita*) [23]; increased the content of monoterpene indole alkaloids in periwinkle (*Catharanthus roseus*) [24]; and led to the accumulation of various terpenoids, such as chlorophyll a, luteolin, β-carotene, and lycopene, in *N. tabacum* [25]. Interestingly, mutations in *DXR* also affect the expression of plastid-encoded genes and inhibit gibberellins (GAs) and abscisic acid (ABA) biosynthesis due to the metabolic disruption of enzyme mutants involved in the MEP pathway [26,27]. These results reveal a critical role for the MEP biosynthetic pathway in controlling the biosynthesis of isoprenoids. DXR rate-limiting enzymes are key controllers of functional gene expression in the MEP synthesis pathway of terpenoids.

To investigate the transcriptional regulatory function of DXR enzymes in the MEP synthesis pathway during growth and development of *P. massoniana*, in this study we validated the function and mechanism of action of a 1-deoxy-D-xylulose-5-phosphate-reductoisomerase. As a highly methylated gymnosperm, *PmDXR* has different expressions in the regulation of the MEP pathway during the development of different organs of *P. massoniana*. Here, we demonstrated that *PmDXR* plays a positive role in abiotic stress. By analyzing the promoter, we found that the *PmDXR* promoter regulates the regulatory elements of stress in the metabolic pathways and multiple stresses, as a key enzyme in the MEP pathway. In summary, these results provide new insight into the molecular regulation of the MEP and MVA pathways during the invasion of pine wilt disease and pine wood nematode.

## 2. Results

### 2.1. PmDXR Identification and Bioinformatics Analysis

**Figure 1:**
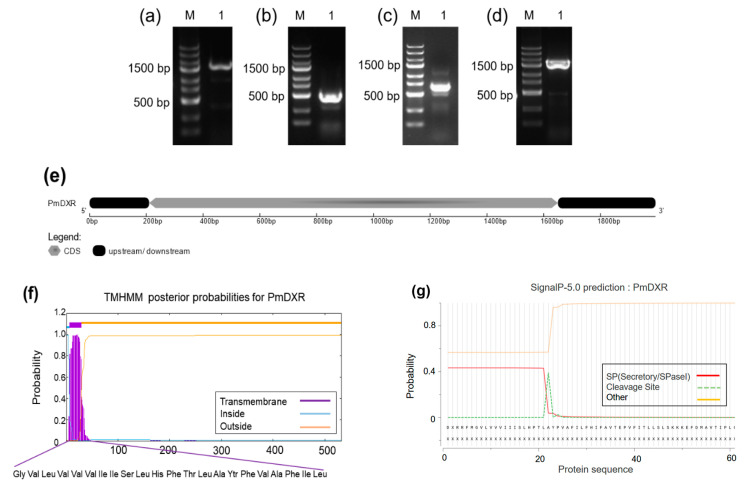

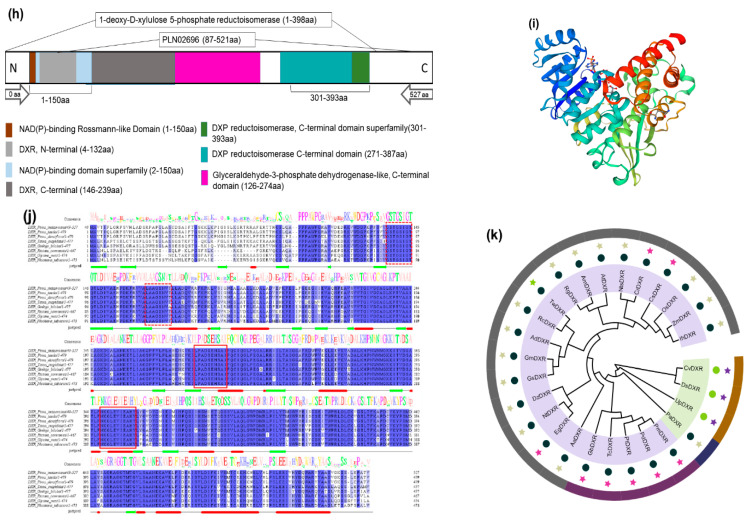
Cloning and structural analysis of the *PmDXR*. (**a**) Product of the intermediate fragment (M: 250 bp-IIDNA ladder; 1: PCR amplification product of the intermediate fragment, 1739 bp). (**b**) Product of the 5′RACE fragment (M: 250 bp-IIDNA ladder; 1: PCR amplification product of the 5′RACE fragment, 357 bp). (**c**) Product of the 3′RACE fragment (M: 250 bp-IIDNA ladder; 1: PCR amplification product of the 3′RACE fragment, 697 bp). (**d**) Product of the ORF fragment (M: 250 bp-IIDNA ladder; 1: PCR amplification product of ORF fragment, 1584 bp). (**e**) Structural features of *PmDXR*. (**f**) Hydrophobicity plot of PmDXR. (**g**) Predicted transmembrane helical structural domains of PmDXR. (**h**) Schematic diagram of the domains of PmDXR. (**i**) Schematic diagram of the tertiary structure of PmDXR. (**j**) Multiple sequence alignments of the deduced amino acid sequences among *PmDXR* and *DXR* from other plants (the DXR functional binding motif is marked by a solid red box, and the NADPH-binding site binding motif is marked with a dashed red box. The “jnetpred” in the figure represents the prediction result of the secondary structure of the DXR protein; the α-helix is represented by a red bar, and the β-sheet is represented by a green arrow). (**k**) Phylogenetic tree analysis of DXR from *P. massoniana* and other species. (Inner circle colors: light purple background represents seed plants, and light green background represents spore plants; outer circle colors: dark purple represents gymnosperms, gray represents angiosperms, brown represents algae, and dark blue represents mosses; asterisk colors: purple represents woody plants, light green represents herbaceous plants, grass green represents lianas, and purple represents algae; dot colors: dark blue for higher plants and green for lower plants. *Pinus densiflora* DXR ACC54558.1, *Pinus taeda* DXR ACJ67022.1, *Taxus cuspidate* DXR AAT47184.1, *Ginkgo biloba* DXR AAR95700.1, *Zea mays* DXR NP_001105139.100.1, *Indosasa hispida* DXR ASU91353.1, *Oryza sativa* DXR AAL37560.1, *Dioscorea zingiberensis* DXR APW35790.1, *Narcissus tazetta* subsp. *Chinensis* DXR ADD82536.1, *Elaeis guineensis* DXR XP_010940050.1, *Arabidopsis thaliana* DXR NP201085.1, *Glycine max* DXR AEB91528.1, *Glycine soja* DXR XP_028231512.1, *Arachis duranensis* DXR XP_015968127.1, *Tripterygium wilfordii* DXR AHW46302.1, *Ricinus communis* DXR XP002511399.1, *Catharanthus roseus* DXR AAF65154.1, *Artemisia argyi* DXR QBB78631.1, *Nicotiana tabacum* DXR NP001312964.1, *Camellia sinensis* DXR AKE33276.1, *Antirrhinum majus* DXR AAW28998.1, *Rehmannia glutinosa* DXR ANW06222.1, *Dunaliella salina* DXR ACT21081.1, *Ulva prolifera* DXR QBP34360.1, *Chlorella variabilis* DXR XP_005850817.1, *Plagiochasma appendiculatum* DXR AFM78686.1, and *Pinus massoniana* DXR MK969119.1).

We cloned and identified a new nucleotide sequence using the 5′RACE and 3′RACE PCR methods and found that it is 1994 bp nucleotides in length (Figure 1e), with a predicted ORF of 1584 bp (Figure 1d), a 3′RACE of 697 bp (Figure 1c) and a 5′RACE of 357 bp (Figure 1b), encoding 527 amino acids, with a predicted molecular weight of 57.43 kD. The PmDXR protein contains 60 total negatively charged residues (Asp + Glu) and 55 total positively charged residues (Arg + Lys) (Table 1). The homology model template for the PmDXR′s tertiary structure construction was 1-deoxy-D-xylulose 5-phosphate reductoisomerase (1 jvs.1. A) with a GMQE value of 0.55 (Figure 1i). This protein has the characteristic sequence of 1-deoxyxylulose-5-phosphate reductase (PLN02696 functional structural domain) and also has the core sequences of the NADB_Rossman superfamily and the DXP_reductoisom functional structural domain (Figure 1h). Amino acids 7 to 29 of the PmDXR protein form a typical transmembrane helix region (Figure 1f). Corresponding to the hydrophobicity predicted by ProtScale, this protein is a hydrophobic protein (Figure 1h) and has an N-terminal signal peptide probability of 3.180% (Figure 1g).

The *PmDXR* had high similarity with the *DXR* of *P. taeda* EU862299.1, *P. densiflora* EU439294.1, and *P. kesiya* var. *Langbianensis* MG764427.1, with a similarity greater than 98%. Multiple comparisons of the DXR amino acid sequences of different species using ClustalX2.1 and Jalview showed (Figure 1j) that the amino acid composition of the DXR functional domains of different species were relatively consistent with two DXR functional binding motifs, LPADSEHSAI and NKGLEVIEAHY; two highly conserved NADPH-binding site binding motifs, GSTGS(I/V)GT and LAAGSN(V/I)T; and the N-terminal proline-rich sequence PPPAWPG(R/T)A. The phylogenetic tree shows (Figure 1k) that PmDXR clusters into a large group with DXRs of other gymnosperms, among which it is more closely related to *P. densiflora* and *P. taeda*, which belong to the same genus *Pinus*.

### 2.2. Codon Preference Analysis of PmDXR

The GC and GC3s of the *PmDXR* gene codons were 43.75% and 34.47%, respectively, indicating that its codons prefer to end in A/T. The results of the CUSP and CodonW calculations (Table 2) showed that 25 codons of the *PmDXR* had RSCU values greater than 1, including 12 codons with RSCU values greater than 1.5 for GCA, GCT, AGA, AGA, GGA, CCA, CCT, TCA, TCT, ACA, TAT, GTT, and AGA, which encodes arginine, and TCT, which encodes serine. The RSCU values of AGA coding for arginine and TCT coding for serine were 3.60 and 2.17, respectively, indicating that a total of 25 codons in the *PmDXR* gene had preferences, of which 12 had strong preferences and 2 had very strong preferences. The RSCU values of the NCG-type codons in *P. massoniana* (GCG: 0.15, CCG: 0.24, TCG: 0.33, and ACG: 0.15) were low, inferring that *P. massoniana* may be a plant with a high degree of methylation.

### 2.3. DXR Gene/Protein Interactions

**Figure 2:**
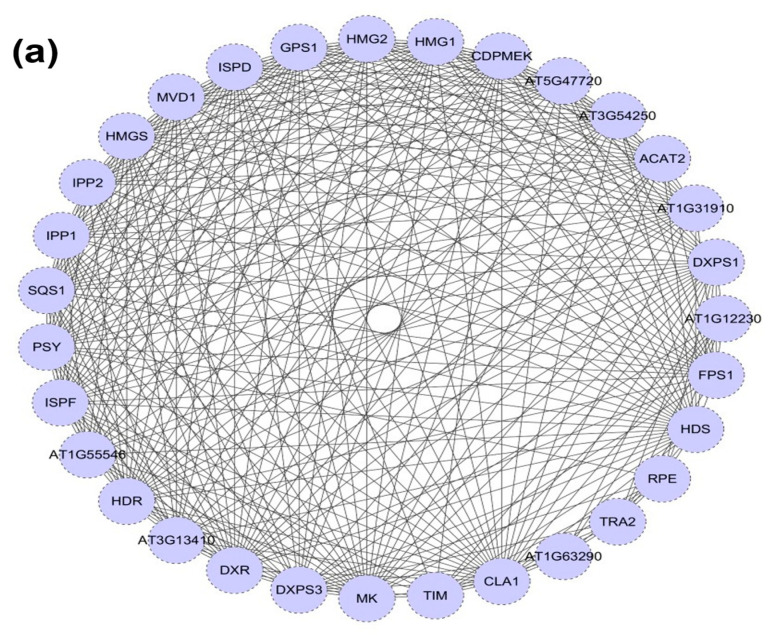

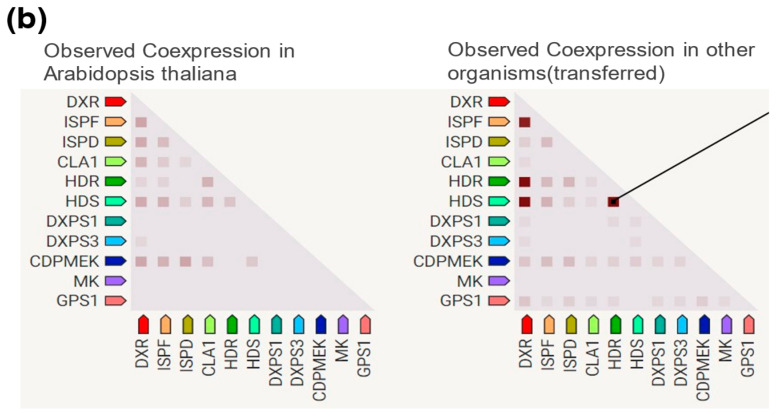
DXS protein interactions with MEP/MVA pathway-related proteins: (**a**) interaction between the PmDXS protein and other pathway related proteins (based on STRING-Toolsearch tool (https://cn.string-db.org/cgi/input?sessionId=bFnV2zuGf1l0&input_page_show_search=on)); (**b**) co-expression of the DXS protein and other interacting proteins (in *Arabidopsis* and other species).

Interactions with proteins related to the MEP and MVA pathways were predicted using *A. thaliana* as a reference species, and a co-expression prediction analysis of DXS of *P. massoniana* with other interacting proteins was performed using *A. thaliana* and other organisms as references (Figure 2a). The PmDXR protein interacted with DXPS, HDR, HDS, IPP, and GPS1 (downstream proteins) in the MEP pathway and with HMGS, HMG, and FPS1 (downstream proteins) in the MVA pathway (Figure 2b). The co-expression analysis with other proteins showed that AtDXR was co-expressed with CDPMEK, HDS, CAL1, DXPS3, ISPD, and ISPF but not with MK and GPS1; *Oryza sativa* and other plants had lower levels of co-expression of DXR proteins with MK proteins. The co-expression study will help in the investigation of differential protein expression and gene regulation in the terpenoid biosynthesis pathway of *P. massoniana*.

### 2.4. Spatial and Temporal Expression Patterns of PmDXR

**Figure 3:**
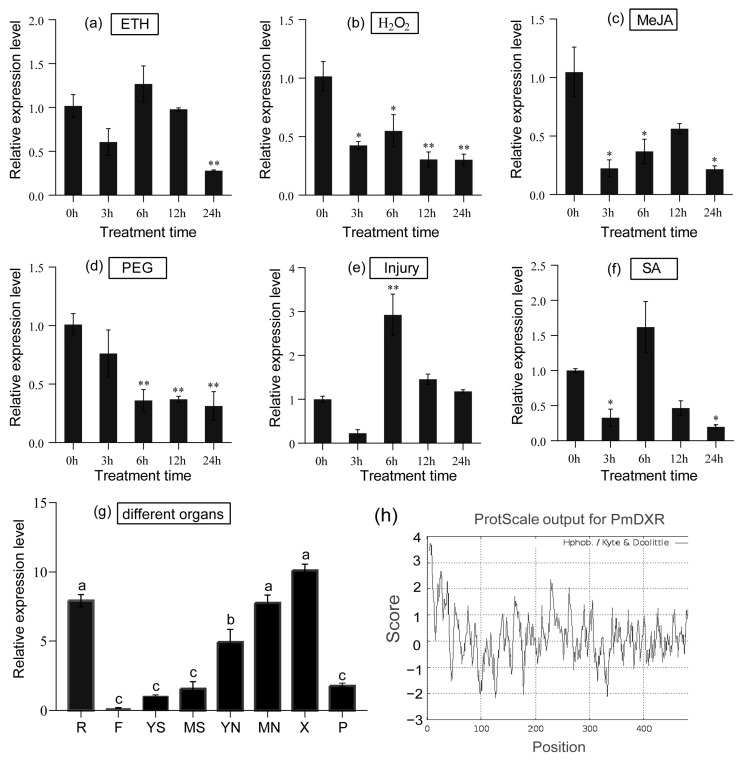
Analysis of the qPCR spatiotemporal expression patterns of *PmDXR*: (**a**–**f**) expression of *PmDXR* under different treatments; (**g**) expression quantity of *PmDXR* in different organs of *P. massoniana* (R: roots; F: flowers; YS: young stems; MS: mature stems; YN: young needles; MN: mature needles; X: xylem; P: phloem). The data represent the mean ± SE of three biological replicates. The asterisks represent significant differences between each treatment time and 0 h (* *p* < 0.05 and ** *p* < 0.01). (**h**) Diagram of the predicted hydrophobicity of the PmDXR protein.

The expression of the *PmDXR* gene in different tissues (roots, young stems, old stems, young needless, mature needles, flowers, xylem and phloem) of *P. massoniana* was analyzed using the qRT-PCR technique. Setting the expression level in young stems to 1, the *PmDXR* expression was highest in xylem, not significantly different from that in mature needles and roots, and significantly higher than that in other tissues. Overall, xylem > roots > mature needles > young needles > phloem > mature stems > young stems > flowers. After mechanical damage treatment (Figure 3e), the expression of the *PmDXR* gene appeared to be upregulated to different degrees at all treatment times, except for 3 h. The expression was highest at 6 h of treatment and was 2.92 times higher than that of the control. After the H_2_O_2_ treatment, MeJA treatment and PEG6000, the expression level of the *PmDXR* gene decreased at all treatment time points (Figure 3b–d). After ETH treatment (Figure 3a), *PmDXR* was slightly upregulated at 6 h of treatment. After the SA treatment (Figure 3f), the expression of *PmDXR* was upregulated at 6 h, and the expression decreased at the rest of the treatment time points.

### 2.5. PmDXR Protein Is Localized to the Chloroplast

**Figure 4:**
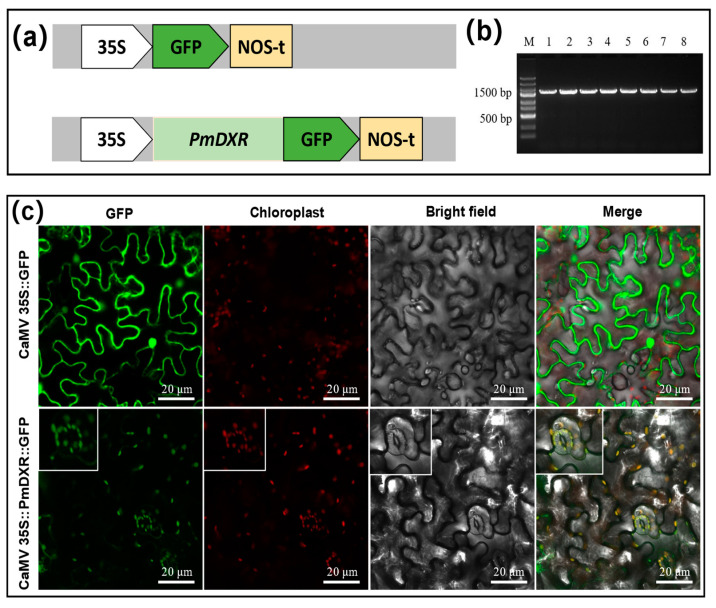
Subcellular localization fusion expression vector construction and GFP fluorescence observation: (**a**) construction of subcellular localization CaMV35S::PmDXR::GFP fusion plasmid; (**b**) detection of CaMV35S::PmDXR::GFP recombinant plasmids in DH5α transformants by bacteriophage electrophoresis (M: DNA marker DL2502; 1–8: CaMV35S::PmDXR::GFP recombinant plasmids, 1994 bp); (**c**) schematic diagram of the observed results of the subcellular localization (GFP: green fluorescence; Chloroplast: chloroplast autofluorescence; Bright field: bright field; Merge: superimposed field).

The CaMV35S::PmDXR::GFP expression vector was successfully constructed using the homologous recombination method (Figure 4a), and the positive single clone was detected by PCR and sequenced correctly (Figure 4b). Transient transformation of *N. benthamiana* by injection and observation of the leaves under laser confocal microscopy revealed that 35S::GFP was localized in the whole tobacco leaf epidermal cells in vacuo, while the CaMV35S::PmDXR::GFP recombinant vector was localized in the chloroplasts of tobacco leaf epidermal cells. We also scanned and captured the chloroplast fluorescence field of a stomatal guard cell, which also confirmed that the N-terminal end of PmDXR has a conserved peptide guiding DXR localization in the plastids (Figure 4c).

### 2.6. Ectopic Expression of PmDXR Promoted Arabidopsis DXR Enzyme Activity and Photosynthetic Pigment Contents

**Figure 5:**
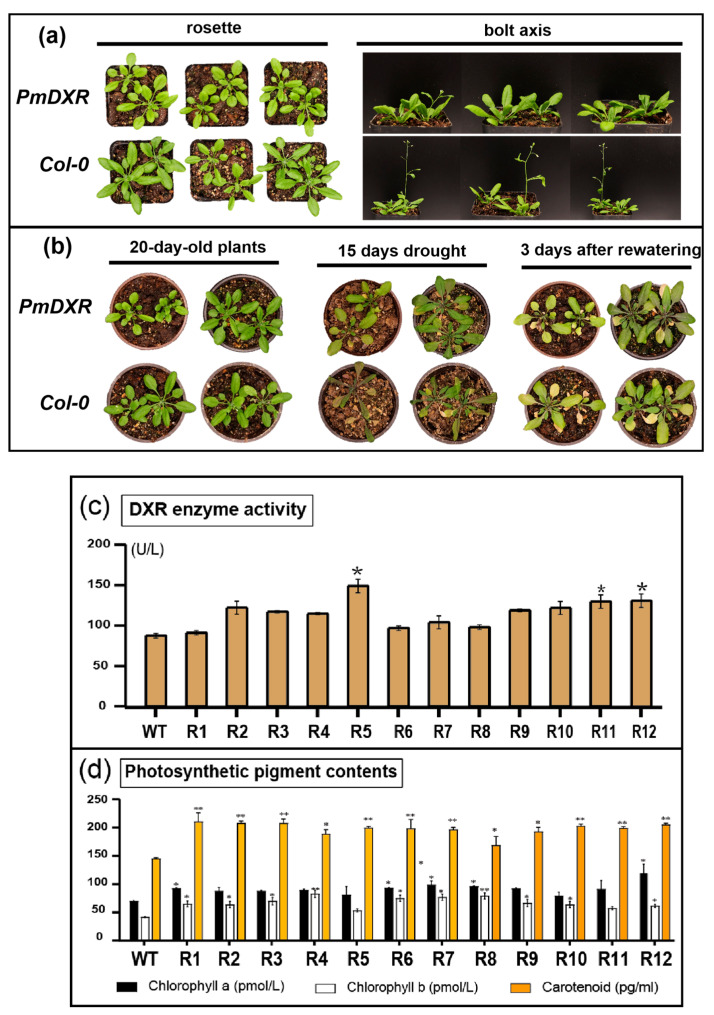
Phenotype observation of transgenic plants and WT plants: (**a**) *Arabidopsis* rosette and *Arabidopsis* bolt axis; (**b**) *Arabidopsis* under drought treatment; (**c**) determination of DXR enzyme activity; (**d**) analysis of photosynthetic pigment contents in transgenic plants and WT Plants. The asterisks represent significant differences between transgenic and wild-type plants (* *p* < 0.05 and ** *p* < 0.01).

Transgenic *Arabidopsis* and wild-type *Arabidopsis* were cultured under the same culture conditions, and the differences in rosette and shoot growth were observed after 30 days of normal growth. There was no significant difference in rosette growth between *Arabidopsis* overexpressing *PmDXR* and wild-type *Arabidopsis*, but there was a difference in carex growth, with the carex of transgenic *Arabidopsis* being significantly lower than that of wild-type *Arabidopsis* (Figure 5a). After 15 days of drought treatment of *Arabidopsis*, it was found that the transgenic *Arabidopsis* grew better than the wild type at 15 days of water deficit, and both transgenic *Arabidopsis* and wild-type *Arabidopsis* could gradually recover after 3 days of rehydration (Figure 5b). We also found that the DXRase activities of the transgenic *Arabidopsis* plants were all higher than those of the wild-type plants, with Line R5, R11 and R12 showing significantly higher DXRase activities than the wild-type plants; R5 showed the highest enzyme activity, reaching 1.7 times that of the wild type, and R11 and R12 showed about 1.5 times the DXRase activity of the wild type (Figure 5c). These results indicate that the transformation of *PmDXR* into *Arabidopsis* elevated the enzymatic activity of DXR. The chlorophyll a, chlorophyll b and carotenoid contents of the transgenic plants increased compared with the wild type, with the chlorophyll a content being 1.1–1.7 times higher than that of the wild type, the chlorophyll b content of the transgenic plants being 1.3–2.0 times higher than that of the wild type and the carotenoid content being 1.2–1.4 times higher than that of the wild type, with Line R1 having the highest carotenoid content of 210.4 pg mL^−1^ (Figure 5d).

### 2.7. Determination of Physiological Indicators of Transgenic A. thaliana under Different Stress Treatments

**Figure 6:**
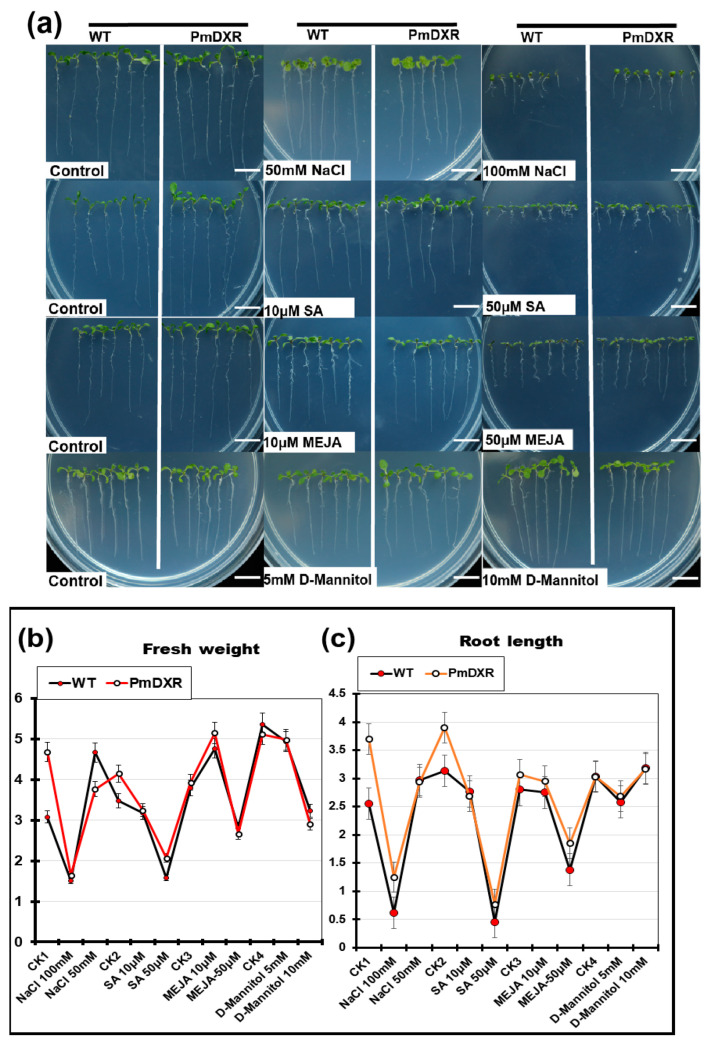
Analysis of phenotypic and physiological indicators of transgenic *A. thaliana* under different treatments: (**a**) phenotype observation of *A. thaliana* under different treatments, scale bar = 1 cm; (**b**) determination of the fresh weight of *A. thaliana* under different treatments (note: CK1-4 is 1/2 MS0); (**c**) determination of the root length of *A. thaliana* under different treatments (note: CK1-4 is 1/2 MS0).

Wild-type and transgenic seeds were spotted on 1/2 MS0 medium and 1/2 MS medium containing different concentrations of NaCl, SA, MeJA and D-Mannitol, and phenotypic changes in *Arabidopsis* were observed and analyzed after 10 days (Figure 6a). The growth of wild-type and transgenic *Arabidopsis* was less affected by the low concentration treatment, and the growth of transgenic roots was less inhibited by the 100 mM NaCl, 50 μM SA and 50 μM MeJA treatments compared with the wild type. However, overall, the root length and fresh weight of the wild-type and transgenic *Arabidopsis* were slightly less affected by each treatment condition (Figure 6b,c).

### 2.8. GUS Staining Analysis of the ProPmDXR Promoter in N. benthamiana

**Figure 7:**
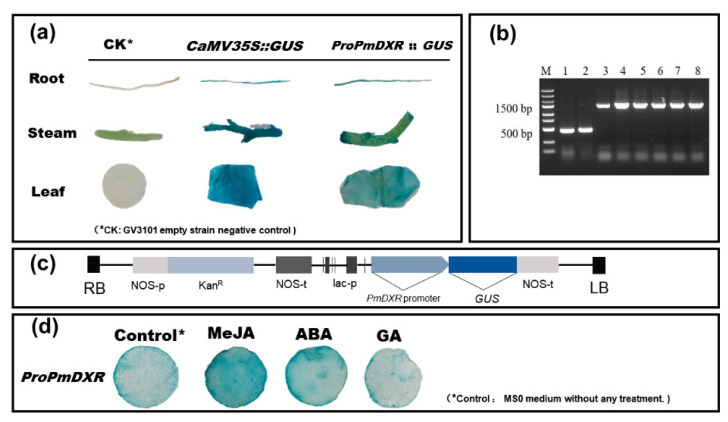
Construction of the PmDXR promoter vector and analysis of GUS activity: (**a**) detection of GUS activity in different tissues; (**b**) PCR detection of pBI121-*proPmDXR*::GUS recombinant plasmid-transformed *E. coli* (M: DNA marker DL2502; 1–2: pBI121 plasmid, 500 bp; 3–8: pBI121-*proPmDXR*::GUS recombinant plasmid, 1600 bp); (**c**) Schematic diagram of pBI121-*proPmDXR*::GUS recombinant vector construction; (**d**) GUS chemical staining analysis of the *PmDXR* promoter under MeJA, ABA and GA.

The 5′ flanking sequence of the *PmDXR* gene, 1600 bp upstream of the start codon (ATG), was obtained after three rounds of PCR amplification and named *ProPmDXR* (Figure 7b). *ProPmDXR* has the basic elements of eukaryotic promoters, TATA-box and CAAT-box, and contains various cis-acting elements, such as the stress response element (STRE), light response element (Sp1 and TCT-motif), jasmonate response element (CGTCA-motif, TGACG-motif) and damage response element (WUN-motif) (Table 3). We successfully constructed the pBI121-*proPmDXR*::GUS fusion expression vector and transiently transformed *N. benthamiana* (Figure 7c). Root, stem and leaf GUS histochemical staining analyses showed (Figure 7a) that the GV3101 null strain of *N. benthamiana*, the negative control, did not have any GUS expression activity, and the roots, stems and leaves transformed with *ProPmDXR* showed different levels of blue color. The results (Figure 7d) of the GUS staining in *N. benthamiana* leaf discs confirmed that the GUS activity driven by *ProPmDXR* after ABA and MeJA treatment was stronger than that in the control (*ProPmDXR* tobacco leaves cultured on MS medium without any hormones). It is hypothesized that *ProPmDXR* is a tissue-specific promoter and an inducible promoter that drives the expressions of GUS reporter genes in tobacco in response to ABA and MeJA stresses.

## 3. Discussion

Turpentine, also known as “the oil that grows on trees”, is more than one-third of the total global turpentine production in China, and it has become a major export commodity of Chinese forestry in the world [28]. As a renewable resource, the utilization of turpentine is beneficial to the sustainable development of the national economy. *P. massoniana* is an important native species, and most of the total annual production of turpentine in China still comes from this species. So, it is crucial to clarify the molecular regulation mechanism of turpentine production to improve the species’ own resistance and increase the national economy. PWD causes pine trees to lose their normal function and the canopy gradually dies, for which no fully effective treatment has been found. It has been shown that an in-depth understanding of terpenoid biosynthesis, such as α-isopinene and β-watercressene, can provide strong support for the development of more effective nematicides, control of PWD, and contribute to forest conservation and economic development [17]. Therefore, it is important to study the synthesis of isoprenoid compounds in plants for the growth and conservation of pine trees. It is especially important to increase their output by improving their yield and regulating the expression of target genes with the help of genetic engineering. In agreement with the results of Li Lu et al. [29], we also hypothesized that the N-terminal end of the PmDXR protein has a conserved peptide guiding DXR localization in plastids, which has been confirmed in the results of subcellular localization, and that the *PmDXR* gene may also be involved in chloroplast formation and play an important role in normal plant growth and development, as well as in the synthesis of secondary metabolites. During the long process of natural selection and evolution, species have developed specific codon usage patterns, and their usage preferences are biologically important for the study of gene evolution and functional identification. The *PmDXR* gene codons prefer more to end in A/T, have a weak codon usage preference, and among the 12 high-frequency codons, AGA and TCT are extremely and strongly preferred, which is consistent with the analysis by Fan Weijun et al. [30], indicating that *P. massoniana* is a highly methylated plant.

DXR regulation of the MEP pathway can vary among plants and their different developmental periods, e.g., *LaDXR* gene expression in *Lavandula angustifolia* correlates with the developmental period of flowering organs [31], and *PdDXR* expression in *P. densiflora* is higher in wood than in needles, bark and roots [19]. The analysis of the gene’s spatiotemporal expression patterns showed that *PmDXR* can be highly expressed in the xylem, mature needles and root tissues of *P. massoniana*, and they are similar to the expression patterns of *Amomum villosum AvDXR* [32], *Populus trichocarpa PtDXR* [33], and *Dioscorea zingiberensis DzDXR* [34]. Terpenoids can be involved in plant defense responses, and phytohormones play an important role in regulating plant growth and development, as well as signaling, but the expressions of *DXR* genes show some variability among species after hormone induction. It was shown that the expression of *PdDXR* increased and then decreased after both 1 mM MeJA treatment and mechanical damage treatment of *P. densiflora* [19]. *Dendranthema indicum DiDXR* could be induced by 0.25% MeJA activation up to a 20-fold expression after 24 h [35]; *GmDXR* expression was significantly higher in *Gentiana macrophylla* leaf tissue after 2 d treatment with the hormone [36]. The expression of *AoDXR* in the rootstock of *Alpinia officinarum* was 1.8-fold higher than the control after 12 h of spraying with 10 μM MeJA [37]. The expression of the *PmDXR* gene in *P. massoniana* needle leaf tissue could be induced by ETH and SA but not by MeJA, which shares the same results with the *Salvia officinalis SmDXR* gene [38] and *Dendrobium officinarum DoDXR* gene [39].

DXR enzymes are involved in the synthesis of secondary metabolites such as pigments in the MEP pathway. When the *DXR* gene is overexpressed, its regulation of the MEP pathway is enhanced, which, in turn, promotes the biosynthesis of terpenoids. Therefore, the content of terpenoids in transgenic plants can be significantly increased by the overexpression of *DXR* genes [40]. We overexpressed the *PmDXR* gene in *Arabidopsis* and found that DXR enzyme activity and chlorophyll a, chlorophyll b and carotenoid contents all increased compared to the wild type. The *PmDXR* gene promoter contains a number of important cis-acting elements that control gene-specific expression, including in response to abiotic stresses and phytohormones. ABA is a very important plant hormone that integrates multiple stress signals to regulate the expression of downstream-related genes under adversity [41]; exogenous MeJA stress induces the expression of plant defense genes and is an important inducer to promote the accumulation of plant secondary metabolites for yield [42]. The *PmDXR* gene promoter responds to ABA and MeJA induction, and CGTCA motif and TGACG motif elements may play a role in response to exogenous MeJA treatment, but the key functional regions regulated in response to MeJA need to be further investigated by constructing deletion bodies, and further studies on the specific regulatory mode of the *PmDXR* gene promoter are needed. In summary, DXR rate-limiting enzymes can directly affect the product production of the MEP synthesis pathway, thereby affecting plant growth and development. These results provide researchers with a new perspective on controlling plant growth and yield by regulating the expression of DXR rate-limiting enzymes. These findings also help us, as researchers, to better understand the complexity of secondary metabolic pathways and gene regulatory networks in plant cells.

## 4. Materials

### 4.1. Pinus massoniana Lamb.

Seeds of *P. massoniana* were obtained from the clonal seed orchard of Baisha State Forestry Farm (latitude: 25°08′58″ N; longitude: 116°35′24″ E) in Shanghang City, Fujian Province, China. For successful germination, seeds were sprouted and rooted in pots containing a mixture of black carbon soil + perlite + vermiculite (4:2:1) and at a relative humidity of 60% to 70%, 25 ± 2 °C, and 16 h light/8 h dark light. Tissue material of 15-year-old seedlings was harvested from the tree garden of Nanjing Forestry University (latitude: 32°47′12″ N; longitude: 118°49′ E) and used for tissue-specific qPCR expression analysis.

### 4.2. Arabidopsis thaliana

The wild-type (Col-0) *A. thaliana* of a Columbia (Col) genetic background was used in this study, and the seeds were preserved by our Lab. Wild-type and transgenic plants were grown under the same culture conditions. *PmDXR* gene transformation was performed by the flush infestation method [43] using 30 mg L^−1^ hygromycin as a screening pressure to screen positive *PmDXR* overexpression plants, which were identified by PCR until T2 generation pure plants were produced. Wild-type and transgenic *A. thaliana* were grown under the same culture conditions. After first sterilizing the surface, wild-type and transgenic *A. thaliana* seeds were spotted on ½ MS medium (20 % sugar, 0.6 % agar and pH 5.85) and then placed in 4 °C dark conditions for 2 days for vernalization and, finally, placed in an artificial climate incubator (25 ± 2 °C, 16 h light/8 h dark light) to wait for germination and growth. After about 7–10 days, the *Arabidopsis* seedlings were transferred to the potting soil with Hoagland nutrient solution (black soil: vermiculite: perlite = 4:2:1) and covered with plastic wrap. The cling film was removed on the third day, the phenotype was observed, and the root length and fresh weight were counted. Each experiment was designed with three replications and at least 90 seeds per genotype. Finally, we took leaves of wild-type and transgenic *A. thaliana* separately and snap-froze them in liquid nitrogen and stored at −80 °C for the extraction of genomic DNA and RNA, a qPCR assay, and an assay of the DXR enzyme activity and photosynthetic pigment content [44].

### 4.3. Nicotiana benthamiana

The seeds of wild-type *N. benthamiana* were stored in our laboratory. After surface sterilization, the seeds were spotted in MS0 medium, and root, stem and leaf tissues were taken after about 21 days for the *PmDXR* promoter *Agrobacterium* transient infestation assay; another portion of seeds were taken in the same manner as *A. thaliana* seed treatment and then transplanted into nutrient soil for 21 days for the PmDXR subcellular localization assay. The environmental culture conditions were the same as those described above for *A. thaliana*, and three biological replicates and three technical replicates were conducted for each set of experiments, with three plants per replicate design.

### 4.4. Reagent

*Agrobacterium* strains EHA105 and GV3101 were purchased from Weidi Biotechnology company (Shanghai, China), and the cloning vectors pEASY^®^-Blunt and *Escherichia coli* strain Transl-T1 were purchased from TransGene Biotechnology company (Beijing, China). The localized expression vector 35S::GFP was obtained from *E. coli* strains preserved in our laboratory.

## 5. Experimental Method

### 5.1. PmDXR Gene Cloning

After searching the *DXR* sequences of different plants in NCBI and comparing them with the Masson pine young shoots transcriptome database (NCBI Accession: PRJNA655997) [45], the target mRNA sequences were screened and the intermediate fragment primers of *PmDXR* were designed using software named PmDXR-MidF and PmDXR-MidR (Table 4). Total RNA of *P. massoniana* was isolated using RNAprep Pure Plant Plus Kit (Tiangen Biologicals, Beijing, China), followed by the removal of genomic DNA contamination using DnaseI Storage Solution (Tiangen). The extracted RNA was reverse transcribed into complementary DNA (cDNA) using the TransScript^®^ One-Step gDNA Removal and cDNA Synthesis SuperMix Kit (TransGen). The RACE cloning primers *PmDXR* 5′RACE Outer, PmDXR 5′RACE Inner, PmDXR 3′RACE Outer, and *PmDXR* 3′RACE Inner (Table 4) were designed with reference to the 3′-Full RACE Core Set with the PrimerScriptRTase Kit (TaKaRa Biologicals, Dalian, China) and the SMARTer^®^RACE 5′/3′ Kit (TaKaRa); reverse-transcribed cDNAs were used for the 3′RACE and 5′RACE cloning templates, respectively. The full-length sequence of the *PmDXR* gene was obtained by splicing using DNASTAR.Lasergene.v7.1 (SeqMan software), and ORF region primers PmDXR ORF-F and PmDXR ORF-R were designed (Table 4).

After the target fragment was obtained, it was ligated by the pEASY-Blunt vector (TransGene Biotech, Beijing, China), transformed with the *Escherichia coli* strain Transl-T1 (TransGene Biotech, Beijing, China), and the sequencing was conducted by the company. The sequences measured were compared with the transcript sequences of our group, and the sequence of the coding region of *PmDXR* was determined. The primers were synthesized by JieRui Biology (Shanghai, China) and sequenced by JieLi Biology (Shanghai, China).

### 5.2. Bioinformatics Analysis

We performed the physicochemical properties and hydrophilicity analysis of the proteins of PmDXR using ProtParam (https://web.expasy.org/protparam/, accessed on 20 April 2020) and ProtScale (https://web.expasy.org/protscale/, accessed on 20 April 2020) online software successively [46]. We also used the online software ProScan (https://www.ebi.ac.uk/interpro/result/InterProScan/, accessed on 20 April 2020) to predict the structural domain of the PmDXR protein. The secondary structure of the PmDXR protein was analyzed using the SOFMA (https://npsa-prabi.ibcp.fr/cgi-bin/npsa_automat.pl?page=npsa_sopma.html, accessed on 20 April 2020) online software [47]. The transmembrane structure of the PmDXR protein was analyzed using TMHMM Server v.2.0 (http://www.cbs.dtu.dk/services/TMHMM/, accessed on 20 April 2020) [48]. The tertiary structure of PmDXR was predicted by Swiss-Model (https://www.swissmodel.expasy.org/, accessed on 20 April 2020) to construct a homology model [49]. The nucleotide sequences of *PmDXR* were compared using Blastn from the NCBI website (https://www.ncbi.nlm.nih.gov/, accessed on 20 April 2020). The multiple sequence alignment of the amino acid sequences was performed using ClustalX2.1 [50] and Jalview [51]. The evolutionary tree based on PmDXR-encoded amino acid sequences was constructed using MEGA5.0 software [52], and the phylogenetic tree was constructed by the neighbor-joining method with the bootstrap method and a test count of 1000; the gaps processing method was complete deletion, and the evolutionary tree was visualized using the online mapping software EvolView (https://evolgenius.info//evolview-v2/#login, accessed on 20 April 2020) [53]. The software CodonW (https://codonw.sourceforge.net/culong.html#CodonW, accessed on 20 April 2020) and EMBOSS (http://emboss.open-bio.org/, accessed on 20 April 2020) and an online server were used to analyze the preference of the codons of *PmDXR*. Protein–protein interactions (PPIs) were predicted and analyzed using the search tool for retrieval of interacting genes/proteins (STRING) analysis (https://cn.string-db.org/cgi/input?sessionId=bop583S4w1aI&input_page_active_form=multiple_sequences, accessed on 20 April 2020) [54] and Cytoscape software (Cytoscape_v3.7.2) [55]. Interactions with proteins related to the MEP and MVA pathways were predicted using *A. thaliana* as a reference species, and the co-expression prediction analysis of PmDXS with other interacting proteins was performed using *A. thaliana* and other organisms as references.

### 5.3. Abiotic Stress and Hormone Treatment of Plant Materials

All tissue samples used for RNA extraction were thoroughly ground in liquid nitrogen. In this experiment, the column purification method was adopted, and the RNA extraction experiment was carried out with reference to the RNAprep Pure Plant Plus Kit (catalogue number DP441, Tiangen Biotech, Beijing, China). The 1st strand cDNA synthesis kit (catalogue number 11141, Yeasen Biotech, Shanghai, China) was used to reverse transcribe RNA into cDNA, and the cDNA was stored at −80 °C.

(1) The relative expression levels of *PmDXR* in young stems (YS), mature stems (MS), young needles (YN), mature needles (MN), flowers (F), xylem (X), phloem (P) and roots (R) of 15-year-old *P. massoniana* were detected.
(2) The expression patterns under abiotic stress and hormone induction of *PmDXR* in 2-year-old *P. massoniana* included the following: mechanic wound, 15% polyethylene glycol (PEG6000), 10 mM H_2_O_2_, 500 µM ethephon (ETH), 1 mM salicylic acid (SA) and 100 µM methyl jasmonate (MeJA). Mechanical damage was treated by cutting the needles at 1/2 of the upper half of the needles in the potted seedlings of *P. massoniana*, and collecting the cut needles at 0 h, 3 h, 6 h, 12 h, 24 h and 48 h intervals. We immediately put the sample in liquid nitrogen and put it in the refrigerator at −80 °C. In addition, the abiotic stress treatment and hormone treatment were carried out by spraying the surface of the plant. The needles of *P. massoniana* were collected every 0 h, 3 h, 6 h, 12 h, 24 h and 48 h, frozen in liquid nitrogen and stored at −80 °C [44,56]. The above treatments were 0 h without any treatment as a control group. Three technical and biological replicates were set up for each group of experiments, and the biological replicates for each treatment consisted of three pots (three plants per pot), with every three seedlings serving as one technical replicate.

### 5.4. Real-Time Quantitative PCR (qRT-PCR) Analysis

The RT-qPCR-specific primers were designed using Primer (premier 5.0) software on the basis of the reference gene sequence, as shown in Table 4, for qPmDXR-F and qPmDXR-R. A StepOnePlus TM Real-Time PCR System with Laptop (Applied Biosystems Inc., Foster, CA, USA) was used to detect the expression of the gene of interest. Hieff UNICON^®^ Universal Blue qPCR SYBGreen Master Mix (Yeasen Biotech, Shanghai, China) was used for qPCR reactions. The *TUA* gene was used as an internal reference gene (NCBI accession number: KM496535.1) (Table 4) [57]. The reference gene *AtActin2* (Table 4) was used for the qPCR analysis of the *PmDXR* gene in *A. thaliana*. The total volume of the PCR reaction was 20 μL: cDNA, 2 μL; forward primer (10 μM), 0.4 μL; reverse primer (10 μM), 0.4 μL; Hieff UNICON^®^ Universal Blue qPCR SYBR Green Master Mix, 10 μL; and sterile ultrapure water, 7.2 μL. The data represent three biological replicates and three technical replicates. The RT-qPCR reaction procedure used was 95 °C for 2 min; 95 °C for 10 s, 55 °C for 30 s, 72 °C for 30 s, 40 cycles, and each reaction was performed in three biological triplicates and three technical replicates.

The relative expression level of the gene was calculated by the 2^−ΔΔCT^method [58] based on the expression level of the gene in the control sample. IBM SPSS Statistics 25.0 (http://www.ibm.com/us/en/, accessed on 5 August 2020) was used for the statistical analysis (*t* test, *p* < 0.05). The plots were created using GraphPad Prism 6 software.

### 5.5. Subcellular Localization of the PmDXR

In order to analyze the subcellular localization of PmDXR, we constructed a GFP fusion expression vector. First, the stop codon of the *PmDXR* gene coding region sequence was removed, and then the digested primers were designed by Snapgene software 6.0 (Table 4; PmDXR-GFP-F and PmDXR-GFP-R). PCR amplification was performed using the pEASY-Blunt plasmid of *PmDXR* as a template to recover the target fragment. We used *Xba*I and *BamH*I to double digest the recovered gene fragment and 35S::GFP empty plasmid [44]. According to the ClonExpress II One Step Kit instructions (Vazyme Biotech, Nanjing, China), the amplified product was connected to the 35S::GFP vector, and the connected product was transferred into *E. coli.* The positive clone was sequenced and confirmed. The recombinant 35S::*PmDXR*-GFP plasmid was transformed into *Agrobacterium* GV3101, and the leaf back of *N. benthamiana* was injected by transient transformation [59]. Subsequently, we used a laser confocal microscope (LSM710, Zeiss, Germany) for fluorescence detection. The experiment was designed with three technical replicates, each containing three seedlings and each injected with three leaves.

## 6. Conclusions

This study identified the key gene PmDXR in the plant isoprene-like synthesis pathway, and its expression level is closely related to the rate of isoprene-like synthesis in plants. On the basis of the transcriptome sequencing data, we identified a second rate-limiting enzyme, PmDXR, in the MEP pathway of terpenoid synthesis from *P. massoniana*, belonging to a plastid protein with a functional structural domain of PLN02696 and two highly conserved NADPH-binding site binding motifs. We speculated that *PmDXR* has spatiotemporal expression specificity in *P. massoniana* xylem, needles and roots and is involved in response to mechanical damage, PEG6000, H_2_O_2_, ETH, SA and MeJA stresses under adversity. Heterologous expression of the *PmDXR Arabidopsis* lines showed negative regulation of the elongation growth of the shoots. The transgenic phenotype showed some sensitivity to growth and development under stress conditions (NaCl, SA, MeJA and D-Mannitol). ABA and MeJA could promote the expression of the *ProPmDXR* promoter, thus enhancing the adaptation of *P. massoniana* to the adversity environment. Therefore, studying the *PmDXR* and its regulatory mechanism in response to adversity is important for understanding the terpene synthesis pathway of *P. massoniana* and its adversity adaptation mechanism.

## Figures and Tables

**Table 1:** The number and proportion of amino acids in the PmDXR protein.

Amino Acid Species	Quantity	Proportion	Amino Acid Species	Quantity	Proportion
Ala (A)	53	10.1%	Leu (L)	51	9.7%
Arg (R)	20	3.8%	Lys (K)	35	6.6%
Asn (N)	9	1.7%	Met (M)	10	1.9%
Asp (D)	26	4.9%	Phe (F)	19	3.6%
Cys (C)	8	1.5%	Pro (P)	34	6.5%
Gln (Q)	10	1.9%	Ser (S)	36	6.8%
Glu (E)	34	6.5%	Thr (T)	26	4.9%
Gly (G)	35	6.6%	Trp (W)	8	1.5%
His (H)	16	3.0%	Tyr (Y)	11	2.1%
Ile (I)	41	7.8%	Val (V)	45	8.5%

**Table 2:** RSCU of the *PmDXR* gene in *P. massoniana*.

Codon	Amino Acid	Proportion	Frequency	Number	Relative Codon Usage	Codon	Amino Acid	Proportion	Frequency	Number	Relative Codon Usage
GCA	Ala	0.377	37.879	20	1.51	CTT		0.235	22.727	12	1.41
GCC		0.189	18.939	10	0.75	TTA		0.176	17.045	9	1.06
GCG		0.038	3.788	2	0.15	TTG		0.196	18.939	10	1.18
GCT		0.396	39.773	21	1.58	AAA	Lys	0.543	35.985	19	1.09
AGA	Arg	0.600	22.727	12	3.60	AAG		0.457	30.303	16	0.91
AGG		0.150	5.682	3	0.90	ATG	Met	1.000	18.939	10	1.00
CGA		0.150	5.682	3	0.90	TTC	Phe	0.316	11.364	6	0.63
CGC		0.000	0.000	0	0.00	TTT		0.684	24.621	13	1.37
CGG		0.100	3.788	2	0.60	CCA	Pro	0.441	28.409	15	1.76
CGT		0.000	0.000	0	0.00	CCC		0.088	5.682	3	0.35
AAC	Asn	0.222	3.788	2	0.44	CCG		0.059	3.788	2	0.24
AAT		0.778	13.258	7	1.56	CCT		0.412	26.515	14	1.65
GAC	Asp	0.385	18.939	10	0.77	AGC	Ser	0.056	3.788	2	0.33
GAT		0.615	30.303	16	1.23	AGT		0.083	5.682	3	0.50
TGC	Cys	0.500	7.576	4	1.00	TCA		0.278	18.939	10	1.67
TGT		0.500	7.576	4	1.00	TCC		0.167	11.364	6	1.00
CAA	Gln	0.600	11.364	6	1.20	TCG		0.056	3.788	2	0.33
CAG		0.400	7.576	4	0.80	TCT		0.361	24.621	13	2.17
GAA	Glu	0.471	30.303	16	0.94	ACA	Thr	0.500	24.621	13	2.00
GAG		0.529	34.091	18	1.06	ACC		0.269	13.258	7	1.08
GGA	Gly	0.400	26.515	14	1.60	ACG		0.038	1.894	1	0.15
GGC		0.143	9.470	5	0.57	ACT		0.192	9.470	5	0.77
GGG		0.200	13.258	7	0.80	TGG	Trp	1.000	15.152	8	1.00
GGT		0.257	17.045	9	1.03	TAC	Tyr	0.091	1.894	1	0.18
CAC	His	0.563	17.045	9	1.13	TAT		0.909	18.939	10	1.82
CAT		0.438	13.258	7	0.88	GTA	Val	0.244	20.833	11	0.98
ATA	Ile	0.390	30.303	16	1.17	GTC		0.089	7.576	4	0.36
ATC		0.146	11.364	6	0.44	GTG		0.222	18.939	10	0.89
ATT		0.463	35.985	19	1.39	GTT		0.444	37.879	20	1.78
CTA	Leu	0.157	15.152	8	0.94	TAA	TER	0.000	0.000	0	0.00
CTC		0.098	9.470	5	0.59	TAG		0.000	0.000	0	0.00
CTG		0.137	13.258	7	0.82	TGA		1.000	1.894	1	3.00

The underlines mean that the value of RSCU > 1.

**Table 3:** Part of the putative cis-acting elements and their positions in the *PmDXR* promoter.

Cis Element	Sequence	Function	Quantity
AT-rich element	ATAGAAATCAA	ATBP-1-binding sites	1
CAAT-box	CAAT/CCAAT/CAAAT	Promoter and enhancer region regulatory elements	30
CAT-box	GCCACT	Syngeneic tissue expression cis-elements	1
CCAAT-box	CAACGG	MYBHv1-binding site	1
CGTCA-motif	CGTCA	Methyl jasmonate response element	1
STRE	AGGGG	Stress response element	8
Sp1	GGGCGG	Light-responsive elements	3
TC-rich repeats	ATTCTCTAAC	Defense and stress response cis-acting elements	1
TCT-motif	TCTTAC	Partial photoresponse elements	1
TGACG-motif	TGACG	Methyl jasmonate response element	1
WUN-motif	AAATTTCTT	Damage response element	1
W-box	TTGACC	Salicylic acid response element	1
TATA-box	ATATAT/TATA/TATATA/ATTATA/ATATAA/TATACA/TATAA/TATATAA/TATATAAATC	Transcription initiation-30 core promoter element	20

**Table 4:** All primers in this experiment.

Primer Name	Sequence of Primers (5′ → 3′)	Use
*PmDXR*-Mid-F	GGTGTCCAATTCCACTACTACATTGC	Gene cloning
*PmDXR*-Mid-R	CATGATAAAAGGCATCCCTTCATGGG	
*PmDXR* 5′RACE Outer	GCGGGAGGAGGTGCTTGTAGGGA	
*PmDXR* 5′RACE Inner	GGAAAGGGGCGGAGGATAAGACAAA	
*PmDXR* 3′RACE Outer	CTGGCCTCGGCTTGACCTTTGCG	
*PmDXR* 3′RACE Inner	GCTTGGAGCCTGCCACAGTCTTC	
*PmDXR* ORF F	ATGGGAGTATTAGTAGTAG	
*PmDXR* ORF R	GACTGTGGCAGGCTCCAAGC	
q*PmDXR*-F	GTTCCCTACAAGCACCTCCTC	qRT-PCR
q*PmDXR*-R	GTTCGGCAACAATGTCCAAT	
*TUA*-F	CAAACTTGGTCCCGTATCCTC	
*TUA*-R	CACAGAAAGCTGCTCATGGTAA	
pPmDXR-SP1	GTGAAGATGGCAGAGTCGCAGGAA	Promoter cloning
pPmDXR-SP2	GCGAGTGTAGGGTGGAGGCTTATT	
pPmDXR-SP3	GGGCGGAGGATAAGACAAAGAAGA	
pPmDXR-F	TGGTAATGCAATGAAGTTGGGAGG	
pPmDXR-R	GGGGTGGAAAGGGGCGGAGGATAA	
pBI121-ProDXR-F	GACCATGATTACGCCAAGCTTTGGTAATGCAATGAAGTTGGGA	
pBI121-ProDXR-R	ACCACCCGGGGATCCTCTAGAGGGGTGGAAAGGGGCGGA	
*PmDXR*-GFP-F	GAGAACACGGGGGACTCTAGAATGGGAGTATTAGTAGTAGTAATAATAATAAG	Subcellular localization
*PmDXR*-GFP-R	GCCCTTGCTCACCATGGATCCGACTGTGGCAGGCTCCAAGC	
1302-*PmDXR*-F	CGGGGGACTCTTGACCATGATGGGAGTATTAGTAGTAGTAATAATAATA	Overexpression of *A. thaliana*
1302-*PmDXR*-R	ACTAGTCAGATCTACCATGGTCAGACTGTGGCAGGCTCCAAG	
1302-CheckF	ACAGTCTCAGAAGACCAAAGGGCA	
*AtActin2*-F	ACTCTCCGCTATGTATGTCGCC	
*AtActin2*-R	ATTTCCCGCTCGCTGTTGTGGT	

## Data Availability

Data are contained within the article.
